# Immune Requirements of Post-Exposure Immunization with Modified Vaccinia Ankara of Lethally Infected Mice

**DOI:** 10.1371/journal.pone.0009659

**Published:** 2010-03-11

**Authors:** Henning Lauterbach, Ronny Kassub, Juliane Pätzold, Jana Körner, Michael Brückel, Admar Verschoor, Paul Chaplin, Mark Suter, Hubertus Hochrein

**Affiliations:** 1 Department of Research, Bavarian Nordic GmbH, Martinsried, Germany; 2 Preclinical Development Department, Bavarian Nordic GmbH, Martinsried, Germany; 3 Institute for Medical Microbiology, Immunology and Hygiene, Technische Universität München, Munich, Germany; 4 University of Zurich, Zurich, Switzerland; National Institute of Allergy and Infectious Diseases, United States of America

## Abstract

Current prophylactic vaccines work via the induction of B and T cell mediated memory that effectively control further replication of the pathogen after entry. In the case of therapeutic or post-exposure vaccinations the situation is far more complex, because the pathogen has time to establish itself in the host, start producing immune-inhibitory molecules and spread into distant organs. So far it is unclear which immune parameters have to be activated in order to thwart an existing lethal infection. Using the mousepox model, we investigated the immunological mechanisms responsible for a successful post-exposure immunization with modified vaccinia Ankara (MVA). In contrast to intranasal application of MVA, we found that intravenous immunization fully protected mice infected with ectromelia virus (ECTV) when applied three days after infection. Intravenous MVA immunization induced strong innate and adaptive immune responses in lethally infected mice. By using various gene-targeted and transgenic mouse strains we show that NK cells, CD4 T cells, CD8 T cells and antibodies are essential for the clearance of ECTV after post-exposure immunization. Post-exposure immunization with MVA is an effective measure in a murine model of human smallpox. MVA activates innate and adaptive immune parameters and only a combination thereof is able to purge ECTV from its host. These data not only provide a basis for therapeutic vaccinations in the case of the deliberate release of pathogenic poxviruses but possibly also for the treatment of chronic infections and cancer.

## Introduction

Prophylactic vaccination, meaning the prevention of an infectious disease by administration of attenuated or killed pathogens or subunits thereof, remains one of the most important measures to maintain public health. The list of vaccine-preventable diseases currently includes 27 diseases, ranging from Anthrax to Yellow Fever (http://www.cdc.gov/vaccines/vpd-vac/default.htm). The large-scale vaccination with live vaccinia virus (VACV) that led to the worldwide eradication of variola virus (VARV), the causative agent of smallpox, is often cited as the most successful vaccination program [1]. However, people born after the cessation of the general smallpox vaccination in the late 1970's are at risk of poxvirus infections. Besides accidental or intentional (bioterrorism) release of VARV, zoonotic poxvirus infections (e.g., monkeypox) also have to be envisaged as potential threats [2]. This has lead to several governments stockpiling traditional smallpox vaccines based on VACV, although the associated side effects of the wide spread use of smallpox vaccines based on replicating VACV [3], [4] probably restrict their use to an emergency or post-exposure situation. Thus, in cases of sudden outbreaks, caused either naturally or through bioterrorism, efficient and fast acting treatments have to become available.

As an alternative to the usage of antibiotics and antivirals to combat existing infections the idea of therapeutic vaccination is becoming increasingly attractive. This approach is currently investigated mainly for the treatment of chronic infections and cancer. The restricted use of traditional VACV smallpox vaccines due to safety concerns, particularly for people with impaired immune systems [5] has led to the development of potentially safer alternative vaccines based on a highly attenuated, non-replicating poxvirus, Modified Vaccinia Ankara (MVA; reviewed in [6], [7]. Recent studies of our group [8] and others [9], [10], have demonstrated the efficacy of post-exposure vaccination in an acute and lethal virus infection model using MVA or ECTV. In this model mice were intranasally infected with ectromelia virus (ECTV), the causative agent of mousepox. The course of disease is very similar for mousepox and smallpox, including the entry route, the high infectivity at low doses, the development of viremia, the restricted host range and the delayed but fatal outcome (reviewed in [11]). Therefore, mousepox can be regarded a valuable small animal model for human smallpox and, in general, as a model for acute, fatal viral diseases.

While many associate the efficacy of prophylactic VACV immunization to be reliant on the induction of antibody responses (for review see [12]), the requirements for a successful therapeutic immunization are not defined at all. We previously showed that ECTV infected C57BL/6, Toll Like Receptor (TLR) 9 deficient and interferon α receptor (IFNAR) deficient, but not recombination-activating gene (Rag) 1 deficient mice could be protected by simultaneous or post-exposure (only TLR9^−/−^) immunization with MVA [8]. These and other data [9] demonstrate that the induction of adaptive immune responses is critical for a successful therapeutic immunization in the mousepox model. Since the essential roles of both innate and adaptive immune responses in the survival of a primary ECTV infection have been well established [13]–[20], we sought to define their respective roles in a therapeutic vaccination protocol.

The highly attenuated MVA has a better safety profile and fewer immunomodulatory molecules than live VACV and is known to induce antibody and T cell responses in mice and humans [6], [7], [21]. Furthermore, it was superior in postexposure immunizations [9]. We therefore used MVA in order to define the immunological requirements for the therapeutic protection of mice from a lethal ECTV infection. Through the use of various transgenic and knock-out mice we clearly demonstrated that only a combination of NK cells, neutralizing antibodies, CD4 and CD8 T cells is able to thwart a lethal ECTV infection after therapeutic MVA vaccination. Crucially, in this model we show that the efficacy of this therapeutic vaccination strongly depends on the immunization route.

## Results

### ECTV infected C57BL/6 mice can be rescued by intravenous post-exposure MVA immunization

Previously we were the first to demonstrate the feasibility of a therapeutic vaccination regimen in an otherwise lethal orthopoxvirus infection [8]. In this case we rescued C57BL/6 mice from a lethal mousepox infection by immediate vaccination with MVA. In addition, we protected highly susceptible TLR9-deficient mice with intranasal MVA immunization even two days after ECTV infection. In this setting all mice survived without any obvious signs of illness. A period of three days between infection and immunization still conferred partial protection. In the light of these promising results we sought to further investigate the potential and the immunological mechanisms of therapeutic vaccination with MVA.

**Figure 1:**
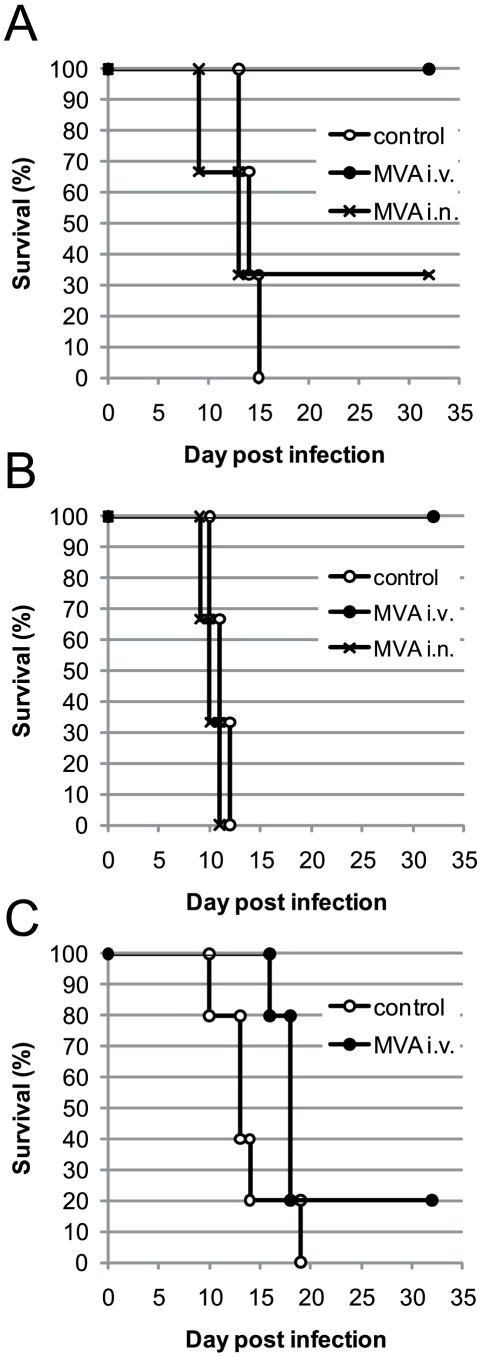
Intravenous post-exposure MVA immunization provides better protection than intranasal immunization. C57BL/6 mice were infected intranasally with 3×10^4^ TCID_50_ of ECTV. Mice were left untreated or immunized after 2 (A), 3 (B) or 5 (C) days with 5×10^7^ TCID_50_ of MVA either intravenously or intranasally. Note, in C no i.n. group was included. Survival was monitored for 32 days. The experiments were performed with 3–5 mice/group. Experiments for A were performed twice, for C once and B shows one exemplary experiment out of three.

First, we wanted to know whether our findings in TLR9-deficient mice, which are 100-fold more susceptible to ECTV infection [8], are transferrable to C57BL/6 wild type mice. C57BL/6 mice were infected with a lethal dose of 30000 TCID_50_ of ECTV (of note, this dose is about 300 times higher than the one used for TLR9^−/−^ mice [8] and corresponds to ∼14 LD_50_) and immunized two days later with MVA. Because ECTV is able to spread from a local infection site to peripheral organs, such as spleen, liver and ovaries, we included a systemic immunization route. Only the systemic intravenous immunization protected all mice from death, whereas only one out of three intranasally vaccinated mice survived (Fig. 1A). If MVA immunization was performed three days after infection, all intranasally immunized mice died with the same kinetics as unvaccinated control mice, but still none of the intravenously vaccinated mice succumbed (Fig. 1B). The intravenous application only was less protective if we waited five days before MVA vaccination and in this case only one out of five animals survived (Fig. 1C). However, mice immunized 5 days after ECTV infection died with delayed kinetics compared to the control group.

### Early activation of innate and adaptive immune responses after intravenous post-exposure MVA immunization

**Figure 2:**
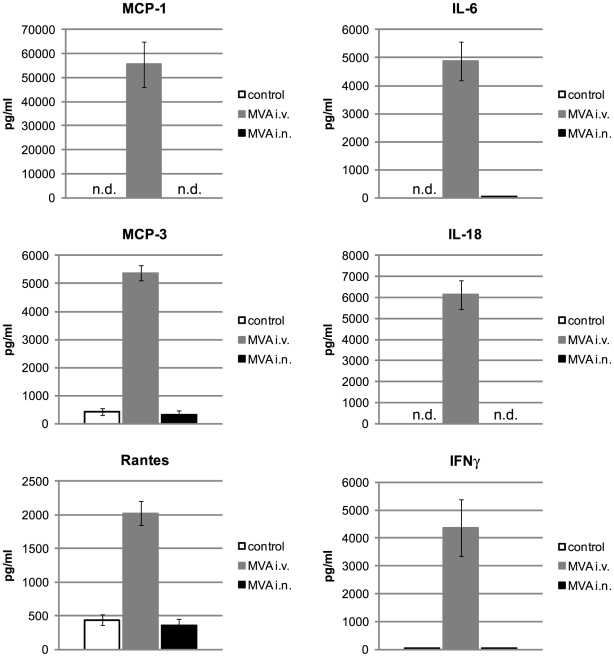
Intravenous but not intranasal post-exposure MVA immunization induces strong systemic cytokine production. C57BL/6 mice were infected intranasally with 3×10^4^ TCID_50_ of ECTV. Mice were left untreated or immunized after 3 days with 5×10^7^ TCID_50_ of MVA either intravenously or intranasally. 6 hours after MVA immunization mice were bled and serum cytokine levels were determined using a bead-based detection assay. Data are means ± SD of 5 mice per group and are representative of three similar experiments. (n.d.  =  not detectable)

Previous experiments of our group and others demonstrated a partial role of innate and a crucial role of adaptive immunity for the survival of ECTV infected mice even after immediate application of MVA [8], [9]. This and the fact that intravenous post-exposure MVA immunization was far more potent than intranasal immunization, prompted us to further investigate the immunological differences between both application routes. All further experiments were performed with a period of three days between ECTV infection and MVA immunization. First, we evaluated the innate immune response by measuring the levels of 19 cytokines immediately after MVA immunization. Six hours after intravenous immunization, high serum levels of MCP-1, MCP-3, Rantes, IL-6, IL-18 and IFN-γ could be detected (Fig. 2). Cytokine levels after intranasal MVA application were either below the detection limit or indistinguishable from the levels in the control group.

**Figure 3:**
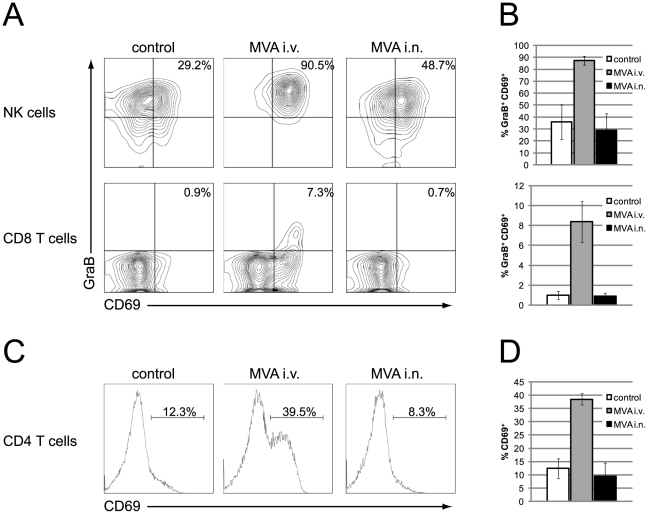
Intravenous but not intranasal post-exposure MVA immunization activates NK and T cells. C57BL/6 mice were infected intranasally with 3×10^4^ TCID_50_ of ECTV. Mice were left untreated or immunized after 3 days with 5×10^7^ TCID_50_ of MVA either intravenously or intranasally. 1 day after MVA immunization mice received an injection of 250 µg BFA and 6 hours later spleens were removed, stained and analyzed by FACS. (A) Representative contour plots are shown for NK cells (NK1.1^+^ CD3^−^) and CD8 T cells (NK1.1^−^ CD3^+^ CD8^+^). The frequency of GranzymeB^+^CD69^+^ NK cells (upper panel) and CD8 T cells (lower panel) is shown in (B). (C) Representative histograms are shown for CD4 T cells (NK1.1^−^ CD3^+^ CD4^+^). The frequency of CD69^high^ CD4 T cells is shown in (D). Data are means ± SD of 4–5 mice per group. The experiment was repeated once with a similar outcome.

Next, we assessed the activation of NK and T cells in the spleen one day after MVA application. In order to directly identify cells that are actively synthesizing granzyme B *in vivo*, we adapted the method developed by Liu et al. [22] and injected 250 µg brefeldin A into all mice six hours prior to intracellular cytokine staining. The activation status was further evaluated by the expression of CD69. In control mice and mice intranasally immunized with MVA about one third of all splenic NK cells expressed high levels of granzyme B and CD69 (Fig. 3A and B). In intravenously immunized mice the frequency went up to 87% (±3.6%). A similar pattern became apparent when monitoring T cell activation: CD69 upregulation (CD4 and CD8 T cells) and granzyme B expression (only CD8 T cells) was only detectable after intravenous, but not intranasal MVA immunization. Thus, intravenous post-exposure MVA immunization induced a rapid strong innate and adaptive immune response as shown by the systemic production of cytokines and the activation of NK and T cells.

### Intravenous post-exposure MVA immunization induces stronger adaptive immune responses than intranasal immunization

Previous experiments have shown that Rag1-deficient mice, lacking mature B and T cells, could not be rescued from lethal ECTV infection by cotreatment with MVA [8], [9]. These findings argued for a crucial role of adaptive immune responses in the post-exposure MVA vaccination model and thus we monitored antibody and CD8 T cell responses in our setting.

In the serum of control mice and intranasally immunized mice, vaccinia-specific IgG levels could first be detected at day 9 post infection (Table 1). At this time point, a neutralizing capacity as measured by a plaque reduction neutralization (PRNT) assay could be detected in both groups. Interestingly, the mouse with the highest IgG and PRNT titer on day 12 in the control group survived, whereas all other mice from those two groups died. In stark contrast, intravenous MVA immunization induced earlier and more vaccinia-specific IgG with higher PRNT titers. Again, all mice from this group survived without any symptoms. Of note, there was a high degree of linear correlation between ELISA and PRNT data (r = 0.93 for day 9 and r = 0.97 for day 12), demonstrating a direct link between antigen-specific IgG titers and the capacity to neutralize live vaccinia virus.

**Table 1:** Geometric mean titers of antibodies.

Group	Mouse	IgG titer (ELISA)			PRNT titer			Day of death
		day 6	day 9	day 12	day 6	day 9	day 12	
**control**	1	1	247	2560	1	6	1289	
	2	1	200	n.a.	1	125	n.a.	14
	3	1	224	1453	1	207	274	13
	4	1	246	n.a.	1	73	n.a.	11
	5	1	100	1495	1	91	221	15
**MVA i.v.**	1	50	1694	7125	1	1875	3774	
	2	50	704	3282	1	485	1493	
	3	50	1640	5538	6	1056	3112	
	4	50	1288	6885	1	963	3508	
	5	50	2863	8524	6	1662	3403	
**MVA i.n.**	1	1	239	1642	1	161	462	17
	2	1	241	n.a.	1	106	n.a.	9
	3	1	n.a.	1015	1	n.a.	535	16
	4	1	100	644	1	91	236	23
	5	1	268	n.a.	1	307	n.a.	11

C57BL/6 mice were infected with 3×10^4^ TCID_50_ of ECTV intranasally. Mice were left untreated or immunized three days later with 5×10^7^ TCID_50_ of MVA either intravenously or intranasally. Anti-MVA IgG titers were calculated by linear regression and defined as the serum dilution that resulted in an optical density of 0.30. Sera with OD value below 0.3 set to titer of 1 (negative). Geometric mean of neutralizing antibody titers by PRNT were determined as serum dilution able to neutralize 50% of the virus. Similar results were obtained in a separate experiment.

n.a. not applicable (mouse was dead or bleeding was not possible).

**Figure 4:**
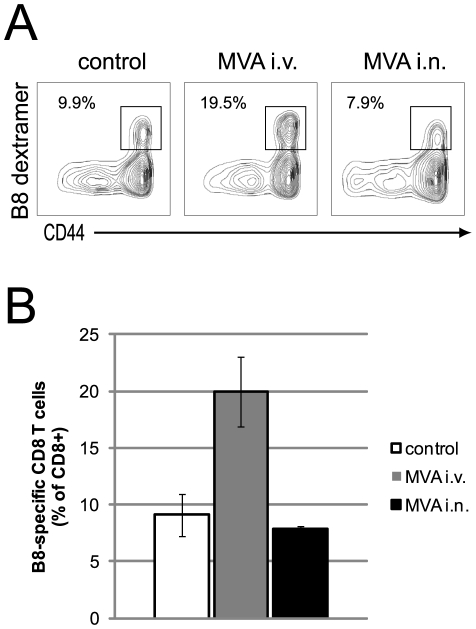
Intravenous post-exposure MVA immunization induces stronger antigen-specific CD8 T cell response than intranasal immunization. C57BL/6 mice were infected intranasally with 3×10^4^ TCID_50_ of ECTV. Mice were left untreated or immunized after 3 days with 5×10^7^ TCID_50_ of MVA either intravenously or intranasally. 5 days after MVA immunization spleens were removed, stained and analyzed by FACS. (A) Representative contour plots are shown for CD8 T cells (CD3^+^ CD8^+^). Numbers show B8-specific cells as percentage of CD8 T cells. (B) The frequencies of B8-specific CD8 T cells are shown for each group as means ± SD of 5 mice per group. Two more experiments showed similar results.

The B8_20–27_ fragment of the soluble IFN-γ receptor B8 of vaccinia virus was identified as the immunodominant CD8 T cell epitope in C57BL/6 mice [23]. The immunodominance of B8_20–27_ has been shown to be conserved amongst different poxvirus species [23]. In addition, peptide immunization with this epitope confers protection against ECTV challenge [23]. In order to evaluate vaccinia-specific CD8 T cell responses, we thus stained spleen cells eight days after ECTV infection with MHC class I multimers loaded with the B8_20–27_ peptide. Again, only intravenous MVA application induced significantly more B8-specific CD8 T cells compared to non-immunized control mice (P≤0.005) (Fig. 4 and B). Intranasally immunized and control mice had the same frequency of splenic B8-specific CTL.

Taken together, these experiments show clearly that after intranasal post-exposure MVA immunization both innate and adaptive immune responses were not enhanced above the threshold already reached with ECTV infection. In stark contrast, intravenous application of MVA three days after ECTV infection efficiently activated and enhanced both arms of the immune system over and above the threshold already reached by the ECTV response. Because we only measured systemic immune responses, we cannot exclude local immune stimulation by intranasal immunization, which, however, would have been insufficient to protect ECTV infected animals in our model.

### Innate and adaptive immune mechanisms are crucial for a successful post-exposure MVA immunization

The above experiments showed that innate and adaptive immune responses already induced by ECTV could be further enhanced by intravenous immunization even when MVA was applied three days after ECTV. Apparently the time window for a successful post-exposure immunization is rather narrow (Fig. 1C), making it likely that the time between immunization and a putative death must be long enough to develop or to enhance adaptive immunity. Previous results in Rag1-deficient and IFNAR-deficient mice [8], [9] suggest that both innate and adaptive immune mechanisms are necessary for the survival of ECTV infected mice. However, it remains unclear which part or parts of the immune system are responsible for a successful post-exposure MVA immunization. In order to further define the individual immune mechanisms we took advantage of several transgenic and knock-out mouse strains.

As intravenous post-exposure MVA immunization was the most effective route in our model, we focused on this route for further studies. In all experiments non-immunized and 3 day post-exposure MVA immunized wild-type mice served as negative and positive controls, respectively. For clearer presentation of the results, groups are ordered according to mouse strains and cumulative results from several experiments are shown for individual mouse strains.

**Figure 5:**
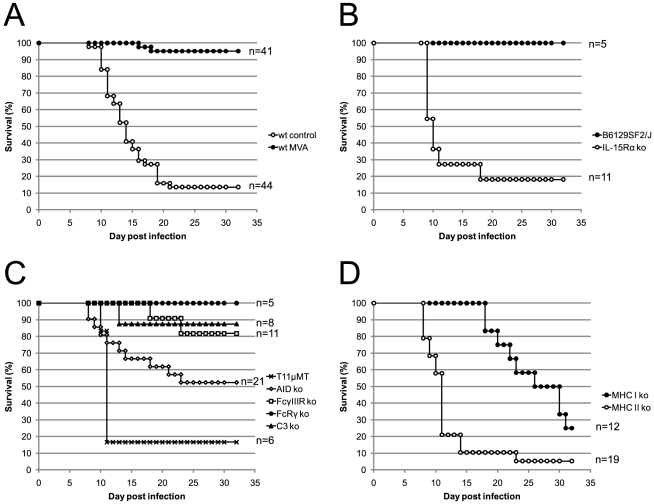
Innate and adaptive immune mechanisms are required for post-exposure protection provided by intravenous MVA immunization. Mice were infected intranasally with 3×10^4^ TCID_50_ of ECTV. (A) Wild-type C57BL/6 mice were left untreated (wt control) or immunized (wt MVA) 3 days later with 5×10^7^ TCID_50_ of MVA intravenously. (B, C and D) All mice were immunized 3 days after ECTV infection with 5×10^7^ TCID_50_ of MVA intravenously. Survival was monitored for 32 days. The experiments were performed with the indicated number of mice, and data show the cumulative results of 10 experiments.

From a total of 44 ECTV infected C57BL/6 mice, 6 animals survived (survival = 13.63%) (Fig. 5A). These animals showed clear symptoms of infection (conjunctivitis, hunched back, ruffled fur, lethargy) between day 8 and 20 post infection and later developed tail lesions and in some cases swollen limbs (data not shown). In contrast, only 2 out of 41 mice in the MVA treated positive control group died (survival = 95.12%). Importantly, none of the surviving animals in this group showed signs of infection at any time point.

### NK cells

NK cells were previously found to be crucial for the early control of ECTV in C57BL/6 mice [20] and were highly activated after intravenous MVA immunization (Fig. 3). Therefore, we wanted to assess the role of NK cells in our model. For this purpose, we took IL-15Rα deficient mice and their corresponding control strain B6129SF2/J. IL-15Rα^−/−^ mice lack NK and NKT cells, but also have reduced numbers of CD8 T cells and γδ intraepithelial lymphocytes [24]. NKT cells, αβ T cells and γδ T cells, however, do not exhibit antiviral function early after ECTV infection [20]. Similar to C57BL/6 mice all MVA treated control mice were fully protected (Fig. 5B). In contrast, 9 out of 11 IL-15Rα^−/−^ mice died within 18 days after infection. Most mice (89%) died between day 9 and 11, suggesting an early role of NK cells for the control of ECTV infection.

### Antibodies and complement cascade

Antibodies seem to be critical for recovery from primary and secondary ECTV infection [13], [25]. In order to define the role of antibodies, we used several mouse strains with individual defects in the generation or functioning of antibodies. First, we used a B-cell receptor transgenic mouse (T11µMT), which can only produce antibodies of the IgM subclass specific to vesicular stomatitis virus (VSV) that is antigenetically unrelated to ECTV [26]–[28]. These mice were used instead of B cell deficient (µMT) mice, because µMT mice show a distorted splenic microarchitecture resulting in a defective T cell response [29], [30]. All but one ECTV infected and MVA immunized T11µMT mice died rapidly between day 10 and 11 after infection (Fig. 5C). These data imply that vaccinia-specific antibodies are needed early during the infection.

In order to further define the required isotypes, we infected AID deficient mice with ECTV and immunized them three days later with MVA. AID deficiency causes a complete defect in class switching, but also leads to a hyper IgM syndrome [31]. We observed a survival of ∼52% with staggered death kinetics (Fig. 5C). In line with previous reports [31] surviving AID^−/−^ mice had no detectable vaccinia-specific IgG1 or IgG2c titers, but showed high IgM titers (data not shown). In order to test whether the IgM containing sera have neutralizing capacity we performed a PRNT assay with pooled sera from four surviving mice. Interestingly, we found a neutralization titer in the range of wild type mice (data not shown). These results imply that the generation of IgG is important, but not essential for a successful post-exposure immunization. High neutralizing IgM titers seem to be at least partly protective in the case where no virus-specific IgG can be generated.

Apart from the direct neutralization of a pathogen, antibodies can activate NK cells and macrophages via Fc-receptors (FcR), a process referred to as antibody dependent cellular cytotoxicity (ADCC). NK cells exclusively express FcγIIIR and in the absence of this receptor NK cells lose their ADCC capacity but still can lyse YAC-1 target cells. Mice deficient in FcγIIIR, were partially protected by post-exposure MVA immunization (∼82% survival) (Fig. 5C), indicating that ADCC can be compensated by other, potentially overlapping, protective mechanisms of antibodies.

Mice lacking the FcRγ chain have defects in expression or signaling of FcεR I and FcγR I, II, III, IV [32]. Despite the pleiotrophic immune defects seen in those mice, they were fully protected with MVA post exposure vaccination (Fig. 5C). These data imply that FcR mediated effects of antibodies are not important in this setting.

Another possibility of antibody mediated virus clearance is the activation of the complement cascade. In order to define the role of the complement cascade for post-exposure MVA immunization we took advantage of C3 deficient mice. C3 is an essential component of the classical, alternative and lectin pathway. Thus, C3^−/−^ mice are unable to activate the cell-killing membrane attack complex and lack the opsonizing function of C3 itself [33]. In our post-exposure setting, however, the complement system seems to play no role because only 1 out of 8 mice died (Fig. 5C).

Taken together, the experiments above demonstrated an essential role for antibodies in post- exposure MVA mediated protection. Antibodies of different isotypes may neutralize virus, activate complement and may interact with Fc receptors. As complement and Fc receptors analyzed singly had no profound effect, these systems may function cooperatively or compensate each other.

### T cells

In a last set of experiments we wanted to define the role of T cells in the survival of ECTV infected and MVA immunized mice. We thus infected MHC class I (β2M^−/−^) and II (H2-Ab1^−/−^) knock out mice and monitored the survival after MVA immunization. MHC class I knock out mice remained healthy for about three weeks and then started to die with a survival rate of 25% (Fig. 5D). In contrast to MHC Class I deficient mice, MHC class II deficient mice already started to die at day 8 post infection and only ∼5% survived (Fig. 5D).

Thus, both T cell subsets participate in the clearance of ECTV after post-exposure MVA immunization, but each subset is required at a different time point after infection.

## Discussion

Due to the successful eradication of VARV and the severe adverse effects associated with VACV vaccination, prophylactic mass vaccination was halted in the 1980s. The current threat of bioterrorism and the possible emergence of human monkeypox have sparked interest in alternative anti-poxvirus therapies. In addition to antiviral pharmaceuticals, vaccination shortly after infection would be such an alternative.

We and others have recently employed the mousepox model in order to investigate the potential of post-exposure immunizations [8]–[10]. ECTV, the causative agent of mousepox, can be naturally found in wild mice. Several isolates have been described since its first discovery in 1929 [34] and ECTV strain Moscow is the most virulent [35], [36]. The pathogenesis of smallpox in humans caused by VARV infection is closely mirrored by mousepox, especially in C57BL/6 mice [10]. Both viruses infect their respective hosts at low doses and cause severe systemic diseases (reviewed in [11]).

Comprehensive studies have focused on the revelation of immune parameters necessary for the survival of primary and secondary ECTV infection. For primary infections, these studies have demonstrated an essential role of innate and adaptive immunity, including TLR9 mediated recognition by DC [8], the complement system [18], NK cells [15], [20], type I and II IFNs [16], [19], B cells [13], [14], CD4 T cells and CD8 T cells [14], [17]. In the case of secondary infections, i.e., after prophylactic vaccination or non-lethal ECTV infections, virus-specific antibodies and B cell memory seem to be sufficient for protection against high dose ECTV challenge [19], [25], [37]. However, in a therapeutic setting, where ECTV has a timely advantage, the requirements might be different. Therefore, we sought to define the immunological mechanisms of post-exposure immunization in the mousepox model. So far, MVA [8], [9], VACV-Lister [9] and ECTV [10] have been used for post-exposure immunizations. Due to its superior safety-profile, the advanced stage in clinical trials and better protective capacity in a post-exposure setting [9] we chose MVA as vaccine.

In our previous study we applied MVA either subcutaneously or intranasally [8]. Paran et al. found different survival rates after intradermal, intramuscular and intranasal immunization [9]. In order to find the vaccination route with the highest protective capacity we compared subcutaneous, intranasal and intravenous MVA application. As subcutaneous vaccination was not protective in our setting when given two days after infection with a lethal dose of ECTV (data not shown) we omitted this route from further experiments. Intranasal immunization was only partially protective when given after two days, but not at all when given three days after ECTV. This contrasts with our findings in TLR9 deficient mice and lower doses of ECTV, where intranasal immunization was completely protective when applied after two days [8].

In the present study, wild-type C57BL/6 mice were infected with 30000 TCID_50_ of ECTV. This infection dose was 300-fold higher than that previously used with TLR9 deficient mice that are at least 100-fold more susceptible to ECTV infection. We hypothesized that this higher dose might have led to earlier virus dissemination and the production of more immune-inhibitory molecules, rendering a local immunization strategy less effective. We envisaged that the superior efficacy of the intravenous application route might be due to the induction of stronger immune responses. Indeed, intranasal MVA immunization did not further enhance the immune response already induced by ECTV (Figs. 1, 2, 3, 4). In stark contrast, intravenous immunization induced strong innate (cytokines, NK cells) and adaptive (CD4 and CD8 T cells, B cells) immune responses.

Among the detected cytokines, IFN-γ has been shown to be crucial for recovery of C57BL/6 mice from mousepox [16]. IL-18 was initially identified as an IFN-γ inducing factor and activates NK cells, which also play a critical role in the control of an ECTV infection [20], [38]. A recent study by Wang et al. found that IL-18 together with IL-12p40 is necessary for ECTV control and recovery from infection [39]. Interestingly, Rantes, IL-18 and IFN-γ promote Th1 responses, the type of immune response that has been implicated in the recovery from orthopoxvirus infections in mice and humans [10], [40], [41]. The chemokine MCP-1 was recently found in the lungs of C57BL/6 mice intranasally immunized with MVA [42]. Lehmann et al. detected this cytokine two days after immunization in bronchoalveolar lavage fluid. We did not detect any MCP-1 in serum of mice six hours after intranasal MVA immunization and this was independent of whether the mouse was ECTV infected or not (Fig. 2 and data not shown). It thus could be that after intranasal application of MVA MCP-1 is produced later and only locally. In a therapeutic setting, where ECTV has three days to replicate, a local production of cytokines would be antagonized by the production of immune evasion molecules. Importantly, five of the six detected cytokines after intravenous immunization can be directly neutralized by the ECTV proteins: viral chemokine binding protein (Rantes, MCP-1 and MCP-3) [43], B8 (IFN-γ) [44] and the viral IL-18 binding protein (IL-18) [45]. Since intranasal MVA immunization is only fully protective in C57BL/6 mice when given within one day of infection [8], [9], we hypothesize that one reason for the lower protective capacity of this immunization route is the neutralization of locally produced cytokines and chemokines by viral binding proteins. Because variola virus encodes a related set of immune evasion molecules, similar neutralization mechanisms are likely to occur in humans after infection with this virus.

In line with the strong systemic cytokine production, we also observed a strong activation of NK and T cells after intravenous MVA immunization (Fig. 3). NK cells and CD4 and CD8 T cells play important roles in the recovery from a primary ECTV infection [14], [15], [17], [20]. In these previous studies mice were infected with a virus dose that is not lethal to wild-type C57BL/6 mice. After infection with a lethal dose, however, these cells are apparently not activated appropriately to eliminate ECTV (Fig. 3). This deficit can be overcome by intravenous but not intranasal post-exposure MVA immunization. Our studies with gene targeted mice proved that all three cell types are important for the recovery from a lethal mousepox infection after post-exposure immunization (Fig. 5). We found an unexpected chronological order in which these cells are required. NK cells and CD4 T cells are essential within the first two weeks of infection. Surprisingly, CD8 T cells become essential only after three weeks. Because MHC class II deficient mice die earlier than MHC class I deficient mice the essential function of CD4 T cells seems not to be the help for a CTL response but either a direct antiviral function, as demonstrated for LCMV [46], or the help for B cells.

The obligatory requirement of B cells and CD4 T cells for the recovery from a lethal ECTV infection was shown by Chaudhri et al. [13]. They used mice that completely lack B cells (B6.µMT) for their studies. However, these mice have multiple defects, ranging from abnormal spleen morphology to reduced numbers of CD4 and CD8 T cells as well as dendritic cells [29], [30]. Also, the roles of B cells as professional antigen presenting cells and antibody producing cells cannot be distinguished. Therefore, we preferred a B cell sufficient mouse model that is unable to produce poxvirus specific serum IgM or IgG (T11µMT) [26]–[28]. In our study these mice died rapidly after 10–11 days (Fig. 5C) and we had similar results with BCR-restricted IghelMD4 mice [47] (data not shown). These data clearly demonstrate that poxvirus specific antibodies are needed early to protect mice from mousepox in a post-exposure setting. The survival data in BCR-transgenic mice are further supported by the IgG serum titers measured by ELISA (Table 1). Again, non-immunized control mice and intranasally immunized mice showed similar titers and kinetics of poxvirus specific IgG. They had detectable IgG levels from day 9 on, which had further increased by day 12. The only mouse that survived from these groups was the mouse with the highest neutralizing antibody titer (control mouse 1, Table 1). All intravenously immunized mice had detectable IgG titers already at day 6. At every time point the titers were higher than in the other two groups and all mice survived.

In order to gain a more detailed insight into the functioning of antibodies, we also performed a neutralization assay (PRNT). PRNT titers followed the same pattern as the IgG titers measured by ELISA. Thus, intravenously immunized mice developed high poxvirus specific IgG titers with neutralizing capacity. Besides neutralization, antibodies can also exert their effects via activation of the complement cascade or ADCC. In survival experiments using C3 deficient mice, we did not find a major role of the complement system for the survival after post-exposure immunization (Fig. 5C). Similarly, mice lacking either the FcRγ-chain or only FcγIII-receptors survived a lethal ECTV infection when immunized three days later with MVA. This suggests that ADCC mediated by NK cells or other Fc-receptor mediated effects may be compensated by other immune elements when analyzed as single missing components. NK cells are most likely required for direct lysing of infected cells within the first few days in the immune response.

The production of antigen specific IgG is important, but not essential in our model since we observed a survival rate of about 50% in AID deficient mice that are unable to switch isotypes. Surviving AID^−/−^ had high poxvirus specific neutralizing IgM titers (data not shown). It is thus possible that the lack of IgG can be partly compensated for by the hyper production of IgM. This also infers that the early death of MHC class II deficient mice cannot be explained solely by the lack of help of CD4 T cells for isotype switching in B cells. Further studies are needed to define the exact role of CD4 T cells in this setting.

In our model intravenous application of MVA was more potent at inducing systemic innate and adaptive immune responses than intranasal application. This superiority was independent of the ECTV infection as seen in vaccination experiments with naïve C57BL/6 mice (unpublished observation). These data are in line with data from immunization experiments done with recombinant MVA either alone or in combination with a DNA prime [48]–[50]. Besides the high immunogenicity of intravenous MVA vaccination these and our data also demonstrate–at least in mice–the safety of this route. The safety of intravenous MVA application was further proven in irradiated rabbits [51]. Interestingly, a replication competent, recombinant vaccinia virus, which is injected intravenously, is currently investigated as an anti-cancer treatment in clinical trials [52]. This treatment was reported to be well tolerated, with only mild systemic toxicity. Even though these promising findings let it seem likely that intravenous MVA immunization of humans could be well tolerated, too, a careful investigation of possible side effects has to be carried out.

**Figure 6:**
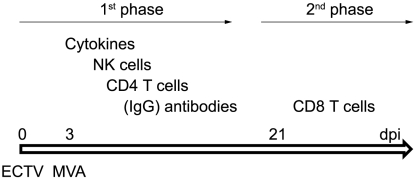
Biphasic mechanisms of post-exposure MVA immunization. In an early phase after ECTV infection (around day 8–20) NK cells, CD4 T cells and antibodies are needed to confer protection after post-exposure MVA immunization. The requirement for IgG can be partly overcome by the production of high amounts of IgM. In a second phase starting around day 21 CD8 T cells become essential. (dpi  =  day post infection)

Our results reveal the immunological mechanisms responsible for the survival of a lethal mousepox infection after intravenous post-exposure immunization. Based on our *in vivo* studies, we suggest the following model (Fig. 6): in a first line of defence antiviral and Th1 inducing cytokines are produced that mediate a systemic antiviral milieu and activate NK cells. NK cells directly target and lyse ECTV infected cells and thus bridge the time until poxvirus specific IgG is produced by B cells. CD4 T cells are needed for B cell help but also for other effector functions. Eventually, when ECTV is repressed by antibodies into the cytoplasm, cytotoxic CD8 T lymphocytes eliminate the remaining infected cells. Thus, the immune requirements involved in post-exposure immunization clearly differ from those involved in prophylactic vaccination, where antibodies and B cell memory alone can provide protection to mice against high dose ECTV challenge [19], [25], [37]. Of note, there is no single parameter that can protect mice from a lethal mousepox infection, but instead only a combination of innate and adaptive immunity can thwart the infection. This comprehensive activation of the entire immune system was only achievable with an intravenous application of MVA. Thus, it is of utmost importance to define the ideal parameters for each vaccine in order to reveal its full potential. This becomes especially important in cases where immediate action has to be taken, such as bioterrorism attacks with VARV. A more efficient induction of innate and adaptive immune responses potentially via the application of existing vaccines via different routes could also be beneficial in cases where standard vaccination regimens have been unsuccessful, so far (e.g., cancer and HIV).

## Materials and Methods

### Ethics Statement

All animal experiments were approved by the government of Upper Bavaria (Regierung von Oberbayern).

### Mice

C57BL/6J mice were purchased from Harlan Winkelmann. MHC class I deficient (B6.129-B2mtm1JaeN12) and MHC class II deficient (B6.129-H2-Ab1tm1GruN12) mice were purchased from Taconic Farms. IL-15Rα deficient (B6;129X1-Il15Ratm1Ama/J), B6129SF2/J and FCγR3 deficient (B6.129P2-Fcgr3tm1Sjv/J) mice were purchased from The Jackson Laboratory. BCR transgenic (T11µMT), AID deficient, C3 deficient and FcRγ deficient mice were obtained from the animal facility of the University Zurich and were on a C57BL/6 background.

### Viruses

The MVA used for this study was MVA-BN®, developed by Bavarian Nordic and deposited at European Collection of Cell Cultures (ECACC) (V00083008). MVA was propagated and titered on primary chicken embryo fibroblasts (CEF) that were prepared from 11-day-old embryonated pathogen-free hen eggs (Charles River, Massachusetts, USA) and cultured in RPMI-1640 medium supplemented with 10% FCS. ECTV strain Moscow was obtained from the American Type Culture Collection (ATCC) as VR-1372, and was propagated and titered on Vero C1008 cells (ECACC 85020206). All cell lines were maintained in Dulbecco's Modified Eagle's Medium (DMEM; Invitrogen) supplemented with 10% FCS without antibiotics. All viruses used in animal experiments were purified twice through a sucrose cushion.

### In vivo experiments

For ECTV infection mice were anaesthetized with ketamine/xylamine and virus (3×10^4^ TCID_50_) was applied by intranasal (i.n.) drop wise installation in a total volume of 50 µl. For i.n. immunizations with MVA mice were anaesthetized likewise. Intravenous (i.v.) injections were given into a lateral tail vein with a total volume of 200 µl. In both cases 5×10^7^ TCID_50_ of MVA were applied. The health status of infected mice was checked daily. For the analysis of granzyme B expression by NK and CD8 T cells mice received an injection of 250 µg brefeldin A (Sigma-Aldrich) one day after MVA immunization. Five hours later animals were sacrificed and spleens were immediately placed into ice cold RPMI/5% FCS medium containing 10 µg/mL brefeldin A. Spleen cells were further processed for flow cytometric analyses.

### Flow cytometry

Spleen lymphocytes were stained after erylysis (RBC lysing buffer, Sigma-Aldrich) using the following monoclonal antibodies: anti-CD3-PECy5, anti-CD3-PECy7, anti-CD4-PerCP-Cy5.5, anti-CD4-APC-H7, anti-CD8-PacificBlue, anti-CD69-FITC, anti-CD44-FITC (all BD Biosciences), anti-NK1.1-PercP-Cy5.5, anti-CD8-Alexa700, anti-IFNγ-PECy7 (all eBioscience) and anti-Granzyme B-PE (Invitrogen). APC-conjugated MHC class I H-2Kb dextramers loaded with B8-peptide (TSYKFESV) were used according to the manufacturers' instructions (Immudex). Intracellular staining of granzyme B was performed after fixation/permeabilization according to the manufacturers' instructions (BD Cytofix/CytopermTM, BD Biosciences). Flow cytometric analysis was performed using a digital LSR II (BD Biosciences). Data were analyzed with FlowJo software (Tree Star).

### Enzyme-linked immunosorbent assay (ELISA)

Vaccinia-specific serum IgG titers were measured on days 6, 9 and 12 after ECTV infection by direct ELISA as described previously [53]. Briefly, 96-well plates were coated overnight with MVA antigen. Test sera were titrated in duplicate using twofold serial dilutions starting at 1∶100. As detection antibody a sheep anti-mouse IgG-HRP (AbD Serotec) was used. The antibody titers were calculated by linear regression and defined as the serum dilution that resulted in an optical density of 0.30.

### Plaque reduction neutralization assay (PRNT)

Vaccinia- and ECTV-specific antibodies were shown to reciprocally neutralize infectivity [54]. In addition, cross protection experiments in mice, rabbits and chicken embryos suggest a high cross reactivity between the two viruses [55]–[57]. In our model, the antibodies are induced partly by ECTV and partly by MVA. Therefore, we decided to use an established Vaccinia-based PRNT assay to determine neutralizing serum antibody levels. The PRNT assay was performed as described earlier [53]. Briefly, heat-inactivated sera were serially diluted and incubated with vaccinia virus Western Reserve (Advanced Biotechnologies Inc., Columbia, MD USA). As a 100% control, virus was incubated with medium only. After incubation the mixtures were added to pre-seeded Vero cells and allowed to adsorb for 70 minutes. After adsorption, pre-warmed medium was added to each well and the plate was incubated for further 24 hours. Visualization of the plaques was performed using a crystal violet solution (Sigma Aldrich). After washing and drying of the plates, plaques were counted. The neutralizing titer was determined as the serum dilution, which was able to neutralize 50% of the mature virus, using the plaque count in the 100% control as 100% value.

### Measurement of cytokines by bead array

Serum cytokine levels six hours after immunization were determined using mouse Th1/Th2 10plex (GM-CSF, IFNγ, IL-1α, IL-2, IL-4, IL-5, IL-6, IL-10, IL-17, TNFα) and chemokine 6plex (GM-CSF, MCP-1, MCP-3, MIP-1α, MIP-1β, Rantes) FlowCytomix Multiplex Kits and IL-13, IL-18, IL-22 and IL-23 FlowCytomix Simplex Kits (Bender MedSystems) according to the manufacturer's instructions. Standard curves were generated and samples quantified using Flow Cytomix Pro 2.2 software (Bender MedSystems). Only MCP-1, MCP-3, Rantes, IL-6, IL-18 and IFNγ were above the level of detection.
